# Impact of culture conditions on oxidative stress responses in HepG2 cells

**DOI:** 10.1016/j.namjnl.2026.100113

**Published:** 2026-07-10

**Authors:** Christina H.J. Veltman, Jeroen L.A. Pennings, Bob van de Water, Mirjam Luijten

**Affiliations:** a Centre for Health Protection, National Institute for Public Health and the Environment (RIVM), Bilthoven, the Netherlands; b Division of Cell Systems and Drug Safety, Leiden Academic Centre for Drug Research (LACDR), Leiden University, Leiden, the Netherlands

**Keywords:** Reactive oxygen species (ROS), Glutathione redox balance (GSH/GSSG), Oxygen tension, Cellular maturation, Repeated exposure

## Abstract

Reliable, mechanism-based *in vitro* assays are essential for Next Generation Risk Assessment of non-genotoxic carcinogens, including those operating through the induction of oxidative stress. This study evaluated how oxygen tension, metabolic capacity and exposure scenarios influence oxidative stress quantification in human hepatocytes, aiming to support the development of *in vitro* assays relevant for chronic toxicity. We compared chemically-induced oxidative stress in HepG2 cells under atmospheric (20% O_2_) and physiological (3% O_2_) conditions by measuring reactive oxygen species (ROS) and the ratio between reduced and oxidized glutathione (GSH/GSSG). Lower oxygen tension reduced cell clustering, slowed proliferation, and mildly decreased hepatocyte marker expression and cytochrome P450 activity. Notably, the potency to induce ROS of most chemicals was lower at 3% oxygen, while the potency to perturb GSH/GSSG was chemical dependent. Maturation of HepG2 cells (mHepG2) yielded a more hepatocyte-like phenotype, with increased expression of metabolic genes and cytochrome P450 activity. Six of the eight tested chemicals showed greater susceptibility to chemically-induced ROS in mHepG2 cells. However, mHepG2 cells had a seemingly better recovery capacity based on the chemical’s potencies to perturb GSH/GSSG. Importantly, mHepG2 cells can be cultured over extended periods, allowing to study the effects of repeated exposure. Repeated exposure with or without recovery periods resulted in increased potency to induce oxidative stress. These findings highlight the importance of oxygen tension, metabolic competence, and exposure frequency for hazard characterization of oxidative stress-inducing chemicals, supporting the development of more predictive *in vitro* assays for chronic toxicity.

## Highlights

• Chemically-induced ROS production is higher under atmospheric oxygen tension.
• Maturation strongly alters HepG2 phenotype and oxidative stress sensitivity.
• mHepG2 cells are suitable for repeated exposure scenarios up to eight days.
• Repeated exposure increases induction of oxidative stress for most chemicals.
• Recovery periods between exposures exacerbate toxic effects of some chemicals.

## 1 Introduction

Risk assessment of chemicals has traditionally relied on animal studies; carcinogenicity testing specifically involves two-year cancer bioassays conducted according to OECD Test Guidelines 451 or 453 (OECD, 2018a, 2018b). However, these animal studies face significant challenges including issues with reproducibility (Gottmann et al., 2001), limited human relevance (Bailey et al., 2014; Marone et al., 2014), high resource- and time-intensity (Schmeisser et al., 2023), and increasing ethical concerns regarding animal welfare (Hendriksen, 2005). In response, the field of toxicology is undergoing a paradigm shift toward the development and application of animal-free approaches. These alternative models, commonly referred to as New Approach Methodologies (NAMs), are designed to provide mechanistic insights into chemical toxicity. NAMs often employ human-derived cells or tissues, which are presumed to yield results that are more relevant to human health (Jaeschke & Ramachandran, 2025). Additionally, NAMs enable high-throughput screening of chemicals, drastically reducing time and resources required compared to traditional regulatory animal studies. Despite their promise in safety testing, however, several limitations remain, including metabolic competence, resembling physiological conditions, and addressing repeated-dose toxicity (Jaeschke & Ramachandran, 2025; Schmeisser et al., 2023; Sewell et al., 2024).

The liver is an important organ in biotransformation and subsequent detoxification of chemicals (Grant, 1991). As such, the liver itself is often targeted by chemical toxicity (Thakur et al., 2024). The considered gold standard to study hepatotoxicity are primary human hepatocytes (PHH) (LeCluyse, 2001). However, these cells come with several issues including limited availability and life-span (Castell et al., 2006). To overcome this, immortalized cell lines are often used in toxicological studies. One of the most commonly used cell lines in hepatotoxicity studies is the HepG2 cell line, derived from human hepatocellular carcinoma. On the downside, HepG2 cells show low cytochrome P450 (CYP) (Boon et al., 2020; Kvist et al., 2018; Pozo Garcia et al., 2025; Wilkening et al., 2003) and solute carrier (SLC) transporter expression (Gerets et al., 2012; Kvist et al., 2018), and altered p53 signaling dynamics (Arzumanian et al., 2021) compared to PHH. As a result, HepG2 cells and PHH differ in sensitivity and mode of cell death after exposure to chemicals such as acetaminophen (Manov et al., 2004; Xie et al., 2015). However, genetically modified HepG2 cells that express CYP2E1 do undergo necrosis following acetaminophen exposure, similar to PHH (Dai & Cederbaum, 1995; Xie et al., 2015). Moreover, HepG2 cells can be maturated to a more hepatocyte-like phenotype using amino acid supplementation (Boon et al., 2020). Maturated HepG2 (mHepG2) cells show improved CYP enzyme activity, and 50% effect concentrations for cytotoxicity following chemical exposure are more similar to PHH compared to non-maturated HepG2 cells (Boon et al., 2020; Pozo Garcia et al., 2025).

Another challenge associated with the use of NAMs is resembling the organ’s physiological conditions. The liver consists of distinct regions with an oxygen gradient between the liver portal tract (± 9%) and the centrilobular hepatocytes (± 4%) (Kietzmann, 2017; Tsukada & Suematsu, 2012). The atmospheric oxygen level (± 20%) generally used for cell culture exceeds the physiological oxygen tension in the liver, increasing the sensitivity of primary mouse hepatocytes to drug-induced cell death due to oxidative stress (Yan et al., 2010). Similarly, HepG2 cells show decreased cell viability following chemical exposure under atmospheric oxygen compared to physiological oxygen conditions (Sakulterdkiat et al., 2012). Oxygen tension can also induce gene expression changes through transcription factors such as hypoxia-inducible factor-1 (HIF-1) and nuclear factor erythroid 2-related factor-2 (NRF2) (Kietzmann, 2019; Ma, 2013). For example, in HepG2 cells arsenic exposure induces heme oxygenase 1 (HMOX1) protein expression under atmospheric oxygen (16%) conditions, whereas under physiological oxygen (5%) levels HMOX1 is already elevated at baseline and arsenic slightly reduces HMOX1 protein expression (Al Taleb et al., 2016). Together these data indicate that chemical effects could be oxygen-dependent, and that responses measured at atmospheric oxygen may not predict physiological effects.

Not only physiological conditions are hard to mimic, the same applies to addressing chronic toxicities following repeated exposure. In humans, drug toxicity typically occurs after weeks or months of exposure to therapeutic doses of the drug (Fontana, 2014). Yet many NAMs only allow for assessment of acute toxicity following chemical exposure, which could lead to underestimation of or complete missing the effect. Nevertheless, newer *in vitro* technologies such as 3D culture and micro-physiological systems allow chemical exposure up to a few weeks (Bell et al., 2016; Hiemstra et al., 2019; Kermanizadeh et al., 2014; Qiu et al., 2023). Notably, these studies show increased sensitivity to chemically-induced cell death upon repeated exposure (Bell et al., 2016; Kermanizadeh et al., 2014), illustrating the importance of addressing repeated-exposure scenarios in the context of chronic toxicity.

A chronic toxicity study, or a study mimicking chronic effects, is considered essential in human health risk assessment of chemicals for carcinogenicity. Within the chemical carcinogens a subdivision has been made based on the chemical’s ability to directly interact with DNA: genotoxic or non-genotoxic (Cohen, 1998). Non-genotoxic carcinogens do not directly interact with DNA, but cause cancer through a variety of mechanisms, including chemically-induced oxidative stress (Saikolappan et al., 2019; Veltman et al., 2023). Two commonly used markers for studying oxidative stress *in vitro* are the production of reactive oxygen species (ROS) and the ratio between reduced and oxidized glutathione (GSH/GSSG) (Frijhoff et al., 2015; Giustarini et al., 2017; Kalyanaraman et al., 2012; Murphy et al., 2022; Reiniers et al., 2021). Reduced glutathione (GSH) serves as a ROS scavenger by donating free electrons to hydrogen peroxide, producing oxidized glutathione (GSSG) and water in the process (Meister, 1988). This reaction is mediated by glutathione peroxidase. To maintain its antioxidant capacity, cells can regenerate GSH through a nicotinamide adenine dinucleotide phosphate (NADPH)-dependent reaction catalyzed by glutathione reductase (Forman et al., 2009; Meister, 1988). Under increased ROS production, more GSH will be consumed and GSSG produced. Normally, the NADPH supply is sufficient to regenerate GSH. However, upon oxidative stress this system may become overwhelmed, leading to GSSG accumulation and an imbalance in the cellular redox status (Forman et al., 2009).

This study aimed to assess the influences of oxygen tension, metabolic maturation and repeated exposure on chemically-induced oxidative stress in HepG2 cells, with the ultimate goal of supporting the development of relevant *in vitro* assays for oxidative stress hazard characterization. For this we applied the DCFH assay, to quantify ROS production directly following chemical exposure, and determined the GSH/GSSG ratio, to study the cellular redox status at the end of chemical exposure. To address potential differences between the conditions without the addition of chemical exposure, we characterized cell morphology, hepatocyte function and oxidative stress status. Additionally, we assessed the viability and stability of mHepG2 cells over time in culture without serum. Relative potency between the different conditions was determined for chemically-induced ROS production and GSH/GSSG ratio perturbation.

## 2 Methods

### 2.1 Chemicals

In this study eight chemicals were assessed for their oxidative stress inducing potential (Table 1). Another four chemicals were used as control chemical (Table 1). All chemicals were obtained from Sigma-Aldrich (Zwijndrecht, the Netherlands). Stock solutions were made in dimethyl sulfoxide (DMSO), except for acetaminophen and sodium arsenite, which were made directly in cell culture medium. A final DMSO concentration of 0.1% was used in all experimental conditions. The concentration range was defined by a combination of the highest achievable soluble concentration and cytotoxicity, and was tested using a logarithmically spaced series.

**Table 1:** Chemicals and concentration ranges tested. ROS = reactive oxygen species formation, GSH/GSSG = ratio in reduced and oxidized glutathione, CYP = cytochrome P450. A final DMSO concentration of 0.1% was used in all experimental conditions. 1 represents the lowest concentration level tested, and 6 the highest.

Chemical	CAS	Mode of action	Concentration range (µM)					
			1	2	3	4	5	6
*Non-carcinogens*								
Acetaminophen (APAP)	103-90-2	CYP2E1-mediated bioactivation and subsequent GSH depletion (McGill & Hinson, 2020; Ramachandran & Jaeschke, 2021)	30	100	300	1000	3000	10000
Diclofenac sodium (DFN)	15307-79-6	Mitochondrial electron transport chain inhibition (Lin et al., 2021)	3	10	30	100	300	1000
Diethyl maleate (DEM)	141-05-9	GSH conjugation and depletion (Comporti et al., 1991)	3	10	30	100	300	1000
Ketoconazole (KCZ)	65277-42-1	Mitochondrial complex I and III inhibition (Haegler et al., 2017)	0.3	1	3	10	30	100
*Non-genotoxic carcinogens*								
Chlorothalonil (CTN)	1897-45-6	Thiol-binding leading to GSH depletion (Yamano & Morita, 1995); mitochondrial complex III inhibition (Slaninova et al., 2009)	0.1	0.3	1	3	10	30
Methapyrilene hydrochloride (MPH)	135-23-9	GSH depletion (Ratra et al., 1998)	3	10	30	100	300	1000
Sodium arsenite (NaAs)	7784-46-5	Mitochondrial uncoupling (Hu et al., 2020)	0.3	1	3	10	30	100
Tebuconazole (TCZ)	107534-96-3	Mitochondrial dysfunction (Petricca et al., 2019)	1	3	10	30	100	300
*Controls*								
N, N’-Diphenyl-p-phenylenediamine	74-31-7	Antioxidant/ Negative control ROS	30					
Hydrogen peroxide	7722-84-1	Positive control ROS	1000					
Menadione	58-27-5	Positive control GSH/GSSG	40					
Dexamethasone	50-02-2	CYP3A4 inducer	50					

### 2.2 HepG2 cell culture, maturation and exposure

HepG2, human hepatocellular carcinoma, cells were obtained from the American Type Tissue Culture Collection (ATCC, Wessel, Germany). Cells were cultured at 37°C and 5% CO_2_ in DMEM high glucose (Gibco, Waltham, MA, USA) supplemented with 10% fetal bovine serum (FBS; Gibco), 25 U/mL penicillin and 25 μg/mL streptomycin (Sigma-Aldrich, Zwijndrecht, the Netherlands). Cell culture was performed at atmospheric oxygen (20%). For more physiological-relevant oxygen conditions, cells were cultured at 3% O_2_ (most representative of the pericentral hepatocytes) for at least two passages before being used in an experiment. Cells were plated in Costar black with clear flat bottom tissue cultured treated 96-well plates (Corning, Kennebunk, ME, USA) at 52,000 (20% O_2_) or 60,000 (3% O_2_) cells per well in culture medium. Cells were allowed to adhere for 72 hours before chemical treatment. Handling of the cells was always performed at atmospheric oxygen.

A previously defined differentiation protocol was applied to mature HepG2 (mHepG2) cells to more hepatocyte-like cells (Boon et al., 2020). In short, cells were plated in Costar black with clear flat bottom tissue culture treated 96-well plates (Corning) at 78,000 cells per well in culture medium. 72 hours after plating, cells were switched to maturation medium (AAGLY) consisting of DMEM low glucose (Gibco) supplemented with 7.7% FBS (Gibco), 20 U/mL penicillin and 20 μg/mL streptomycin (Sigma-Aldrich), 160 μL/mL MEM non-essential amino acids solution (100x; Gibco), 80 μL/mL MEM essential amino acids solution (50x; Gibco) and 2% glycine (Sigma-Aldrich). AAGLY medium pH was adjusted to 7.2-7.4 using sodium hydroxide. Cells were matured for 28 days before starting chemical treatment by renewing medium twice a week. All experiments were performed with cells between passage 12-19.

**Fig. 1:**
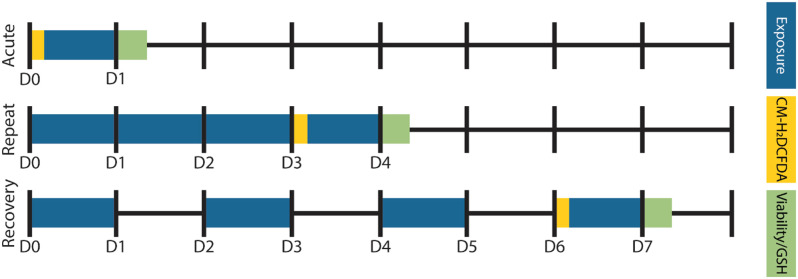
Schematic overview of exposure scenario’s experimental set-up. Three exposure scenarios were tested: acute (1×24 hrs), repeat (4×24 hrs) and recovery (4×24 hrs with 24 hrs in between). Blue blocks represent 24 hours of chemical exposure. Green boxes represent end-point assays for viability and glutathione status. Yellow boxes indicate ROS quantification (live cell assay). D0-D7 = days in serum-free medium.

In total, two different oxygen percentages for HepG2 and three different chemical exposure scenarios for mHepG2 were tested (Fig. 1). In all scenarios, cell culture medium was replaced with freshly diluted chemical in serum-free medium. For acute exposures, cells were treated for 24 hours. In the repeat scenario, cells received daily treatments of 24 h with freshly diluted chemical solution for four consecutive days. For the recovery scenario, cells received four daily treatments of 24 hours alternated with 24 hours of recovery in between.

### 2.3 RNA isolation and real-time quantitative PCR

Total RNA from 20% O_2_ HepG2 cells, 3% O_2_ HepG2 cells and mHepG2 cells was isolated each from 6 wells of a 96-well plate using QIAzol (Qiagen, Hilden, Germany) and the RNeasy mini mRNA isolation kit (Qiagen) according to the manufacturer’s protocol. Quantity of the isolated RNA was checked using the NanoDrop^TM^ 1000 spectrophotometer (Nanodrop Technologies, Wilmington, DE, USA) and quality using the Agilent 2100 Bioanalyzer (Agilent Technologies, Amstelveen, the Netherlands). The High-Capacity cDNA Reverse Transcription Kit (Applied Biosystems, Foster City, CA, USA) was used to convert isolated RNA to cDNA for real-time quantitative PCR. TaqMan gene expression assays (Applied Biosystems) with FAM-MGB dye were used for qPCR (Table 2). The TaqMan™ Fast Universal PCR Master Mix (2X) no AmpErase™ UNG (Applied Biosystems) and fast qPCR program were used in the qPCR reaction, which consisted of 40 cycles of denaturation (95°C for 20 sec), annealing (95°C for 1 sec) and extension (60°C for 20 sec). The qPCR reaction was run on a QuantStudio 6 PCR system using QuantStudio software (version 1.7.2). The 2^−ΔΔCT^ method was used to quantify relative gene expression profiles using *Hprt1, Gusb* and *Polr2a* as housekeeping genes and 20% O_2_ HepG2 cells as reference sample.

**Table 2:** TaqMan gene expression assays used. All primers were bought from Applied Biosystems and use a FAM-MGB dye.

TaqMan assay ID	Gene	Marker
Hs00165475_m1	*Serpina1*	Hepatocyte maturity
Hs00230853_m1	*Hnf4α*	Hepatocyte maturity
Hs00153120_m1	*Cyp1a1*	Metabolism
Hs00167927_m1	*Cyp1a2*	Metabolism
Hs00164383_m1	*Cyp1b1*	Metabolism
Hs03044634_m1	*Cyp2b6*	Metabolism
Hs00426397_m1	*Cyp2c9*	Metabolism
Hs00559367_m1	*Cyp2e1*	Metabolism
Hs00604506_m1	*Cyp3a4*	Metabolism
Hs02511055_s1	*Ugt1a1*	Metabolism
Hs00161820_m1	*Slc10a1*	Bile transport
Hs01110250_m1	*Hmox1*	NRF2-pathway
Hs00544822_m1	*Maff*	NRF2-pathway
Hs00155249_m1	*Gclc*	NRF2-pathway
Hs00168547_m1	*Nqo1*	NRF2-pathway
Hs00607800_m1	*Srxn1*	NRF2-pathway
Hs00232352_m1	*Nfe2l2*	NRF2-pathway
Hs00828652_m1	*Txnrd1*	NRF2-pathway
Hs00153153_m1	*Hif1α*	Hypoxia-pathway
Hs00900055_m1	*Vegfa*	Hypoxia-pathway
Hs00951083_m1	*Tfrc*	Hypoxia-pathway
Hs2800695_m1	*Hprt1*	Housekeeping gene
Hs00939627_m1	*Gusb*	Housekeeping gene
Hs00172187_m1	*Polr2a*	Housekeeping gene

### 2.4 Cytochrome P450 activity

Activity of cytochrome P450 3A4 (CYP3A4) were determined in 20% O_2_ HepG2 cells, 3% O_2_ HepG2 cells and mHepG2 cells using the P450-Glo IPA-assay (Promega, Leiden, the Netherlands). To induce CYP3A4 activity, cells were treated with 50 μM dexamethasone for 24 hours. Both treated and untreated cells were washed once with Dulbecco’s phosphate buffered saline (DPBS) before adding luciferin pro-substrate in serum-free medium. To inhibit CYP activity, luciferin pro-substrate with 1 μM ketoconazole was added to specific wells. After 1 hour of incubation, medium was transferred to a Nunc™ MicroWell™ 96-Well, Nunclon Delta-Treated, Flat-Bottom Microplate (Thermo Fisher Scientific, Roskilde, Denmark) and an equal volume of luciferase reagent was added to the wells. Luminescence was read using a Tecan Infinite M Plex plate reader. The luminescent signal was corrected for medium interference using wells without cells and normalized to total protein content using the Pierce^TM^ BCA protein assay kit (ThermoFisher Scientific). P450 activity was expressed as fold change compared to 20% O_2_ HepG2 cells or 20% O_2_ mHepG2 cells.

### 2.5 Serum-free survival using Alamar Blue

To determine the viability of cells cultured in absence of serum, the Alamar Blue cell viability assay (Invitrogen, Carlsbad, CA, USA) was used. As a positive control, cells were exposed to 100 µM rotenone for 24 hours. Cell viability was assessed daily up to eight days after switching to serum-free medium. Cells were washed once with DPBS before incubation with resazurin solution for 1 hour at 37°C, protected from light. Fluorescence was measured at ex530/em590 nm using a SpectraMax M2 plate reader. The fluorescence signal was corrected for medium interference using wells without cells. Viability was expressed as percentage of the vehicle control (0.1% DMSO).

### 2.6 Cytotoxicity using LDH release

Lactate dehydrogenase (LDH) release was used as measure of cytotoxicity in HepG2 cells cultured under 20% and 3% oxygen after 24 hours of exposure, using a cytotoxicity detection kit (Roche, Woerden, The Netherlands). Cells were lysed in 1% Triton for five minutes before collection of supernatants as positive control for LDH release. Samples were diluted 10X using Dulbecco’s phosphate buffered saline (DPBS) before analysis. Absorbance was measured at 490 nm using a SpectraMax M2 plate reader and corrected for reference wavelength absorbance (650 nm). Absorbance values were corrected for medium interference using wells without cells and background LDH release using the vehicle control (0.1% DMSO) wells. Cytotoxicity was expressed as percentage relative of the positive control, which represents the maximal cytotoxic effect. Cytotoxicity results were used to distinguish chemically-induced effects on oxidative stress from those effects caused by profuse cytotoxicity.

### 2.7 Cell viability using live protease activity

CellTiter-Fluor (Promega) was used as measure of cell viability in HepG2 and mHepG2 cells cultured under 20% oxygen. In short, cells were washed once with DPBS following chemical exposure. To avoid medium interference in the fluorescence measurement, FluoroBrite medium (Gibco) was added to each well. Fresh assay reagent was made and added to the wells in equal volume. Plates were incubated for 30 minutes at 37°C, protected from light. Fluorescence was measured at five locations within a well at ex380/em505 nm using bottom reading on a Tecan Infinite M Plex plate reader. The fluorescence signal was corrected for medium interference using wells without cells. Viability was expressed as percentage of the vehicle control (0.1% DMSO). Cell viability results were used to distinguish chemically-induced effects on oxidative stress from those effects caused by excessive cell death.

### 2.8 Reactive oxygen species quantification

ROS formation was quantified using the fluorescent CM-H_2_DCFDA probe (Invitrogen). CM-H_2_DCFDA was dissolved in DMSO and diluted in Hank’s buffered salt solution (HBSS; Gibco) to obtain a final concentration of 10 μM. Cells were washed with DPBS once before incubation with CM-H_2_DCFDA for 30 minutes at 37°C. After incubation cells were washed once with DPBS and fluorescence was measured at five locations within a well at ex485/em535 nm using bottom reading on a Tecan Infinite M Plex plate reader before starting chemical treatment. After 1 hour, exposure medium was removed, and cells were washed once with DPBS before measuring fluorescence again. The fluorescence signal was corrected for medium interference using wells without cells. The average fluorescence per well was used for calculating ROS formation over time due to chemical treatment. Chemical ROS induction was normalized to the pre-exposure measurement and corrected for untreated (vehicle control) cells: ROS = (T_1_ – T_0_)/T_0_ and ΔROS = ROS_chemical_ – ROS_vehicle control_. To enhance interpretation and comparison across conditions, data was normalized to the positive control: relative ΔROS (%) = (ΔROS_chemical_/ΔROS_positive control_) * 100.

For the repeat and recovery exposure scenario, ROS was determined after 1 h of starting the final exposure interval. During probe incubation, cells were stained with Hoechst 33342 (Sigma-Aldrich) at a concentration of 0.1 μg/mL to visualise the nuclei. Hoechst signal was measured at five locations in each well at ex350/em460nm using bottom reading on a Tecan Infinite M Plex plate reader. A Hoechst filter of < 50% of the plate average was applied to exclude those wells which had significant cell loss. Chemically-induced ROS was normalized to the untreated (vehicle control) cells and expressed as relative change compared to the acute exposure scenario: ROS = chemical/vehicle control and ΔROS (%) = (ROS_repeat_/ROS_acute_) * 100.

### 2.9 Quantification of the ratio between oxidized and reduced glutathione

At the end of the exposure scenarios, cells were lysed and analysed for the ratio between oxidized and reduced glutathione using the GSH/GSSG-Glo kit (Promega) according to the manufacturer’s protocol. In short, exposure medium was removed, and cells were washed once with DPBS before adding total or reduced glutathione lysis buffer. Lysed samples were diluted with equal volume MilliQ to keep samples within the linear range of the assay. Diluted samples were transferred to a Nunc™ MicroWell™ 96-Well, Nunclon Delta-Treated, Flat-Bottom Microplate (ThermoFisher Scientific) and an equal volume luciferin generation reagent was added. Plates were incubated at room temperature for 30 minutes before adding luciferin detection reagent. Luminescence was read using a Tecan Infinite M Plex plate reader. The ratio between total and oxidized glutathione was calculated as follows: ratio = (total – GSSG)/(0.5*GSSG).

### 2.10 Data analysis and statistics

For all experiments three independent biological replicates were performed, except for basal ROS production for which four to six independent biological replicates were performed. Additionally, for cytochrome P450 activity, CellTiter and DCFH, each biological replicate consisted of two to three technical replicates (separate wells). For AlamarBlue, each biological replicate consisted of three to six technical replicates (separate wells). Data is presented as the mean and error bars represent the standard deviation. Statistical significance for qPCR and cytochrome P450 activity was determined using two-way ANOVA with post-hoc unpaired T-test using the Benjamini and Hochberg false discovery rate correction for multiple testing in GraphPad Prism (version 10.5.0). Statistical significance for AlamarBlue, basal ROS and basal GSH/GSSG ratio was determined using one-way ANOVA with post-hoc unpaired T-test using the Benjamini and Hochberg false discovery rate correction for multiple testing in GraphPad Prism. *p_adj_ < 0.05, **p_adj_ < 0.01, ***p_adj_ < 0.001, ****p_adj_ < 0.0001.

Concentration-response data for the various experimental conditions were analysed using nonlinear regression in R (version 4.5.1), using the drc package (version 3.0-1). For each experimental condition, a four-parameter log-logistic (LL.4) model was fitted to the data. Only those experimental conditions with an adequate fit (lack-of-fit p-value > 0.05) were included in further analysis. To determine whether a concentration-dependent response was present, specific criteria were applied to the different assays: a response was defined as a ≥ 20% decrease in cell viability, a fold change of ≥ 1.5 for ROS, or a fold change of ≤ 0.67 for GSH/GSSG, respectively, when comparing the highest to the lowest tested concentration indicated a response. These thresholds were chosen to ensure that only biologically relevant effects were considered in the concentration-response modelling. To assess the uncertainty in model fits, a non-parametric, stratified bootstrap approach was employed using the boot package (version 1.3-31). 1,000 bootstrapping iterations were performed by resampling the data with replacement within each experimental stratum, defined by the combination of experimental condition and concentration. Any bootstraps that failed to converge or produced nonpositive estimates were excluded from the summary statistics. Relative potency (reference/test) between experimental conditions was determined for defined effect sizes: 50% decrease in viability, 20% increase in ROS relative to the positive control, and 33% decrease in GSH/GSSG. Data are presented as the log2-transformed median and 90% bootstrap percentile interval. Data visualization was performed in GraphPad Prism.

## 3 Results

### 3.1 Oxygen tension has minimal effects on the phenotype of HepG2 cells

**Fig. 2:**
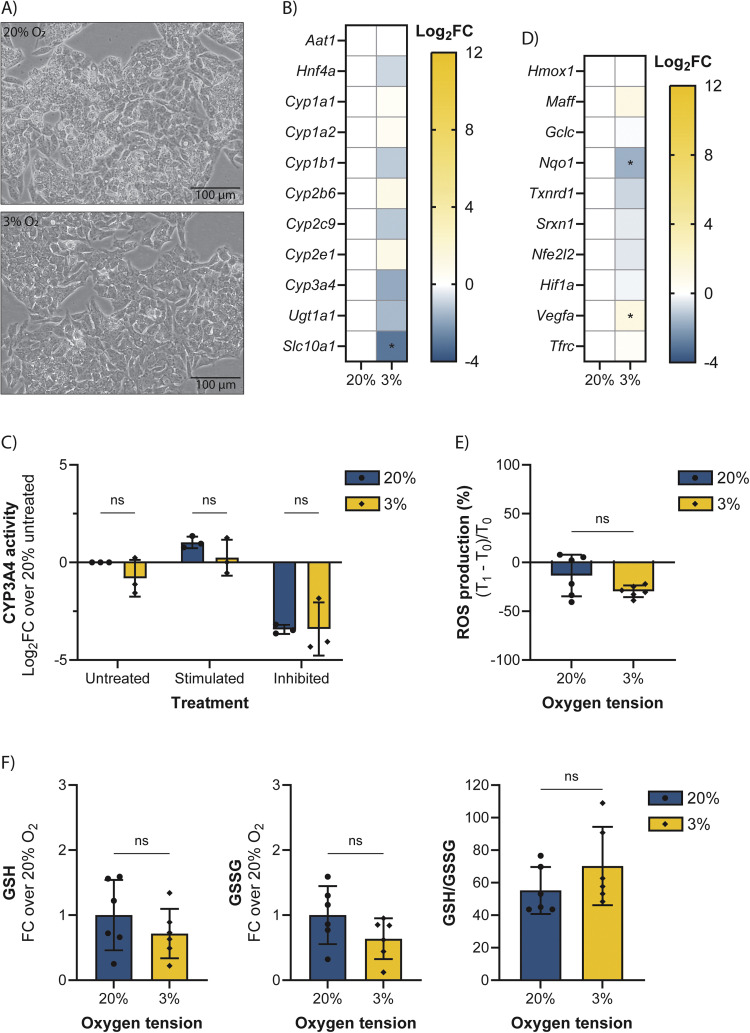
Oxygen tension slightly affects HepG2 phenotype. A) Representative brightfield images of HepG2 cells cultured under 20% oxygen (left) and 3% oxygen (right) using a 10X magnification. Scale bar = 100 µm. B) Relative gene expression (log_2_) changes of hepatocyte markers in HepG2 cells cultured under 3% oxygen compared to 20% oxygen. C) CYP3A4 activity in HepG2 cells cultured under 20% or 3% oxygen. Stimulated cells were treated with 50 µM dexamethasone for 24 hours. Inhibited cells were co-incubated with 1 µM ketoconazole. Log2-fold change compared to 20% oxygen, untreated HepG2 cells. D) Relative gene expression (log_2_) changes of NRF2- and hypoxia-pathway related genes in HepG2 cells cultured under 3% oxygen compared to 20% oxygen. E) ROS production over 1 hour in HepG2 cells cultured under 20% or 3% oxygen. F) GSH, GSSG and GSH/GSSG ratio in HepG2 cells cultured under 20% or 3% oxygen. For GSH and GSSG, fold change compared to 20% oxygen. N = 6. Data are represented as mean ± standard deviation. Statistical significance was determined using a Welch’s T-test. For panels B, D: N = 3. Statistical analysis was performed using a two-way ANOVA followed by post-hoc unpaired t-tests corrected for multiple testing using the false discovery rate (FDR) method: * < 0.05.

Before assessing the effect of chemical exposure on oxidative stress, we assessed the effect of oxygen tension on morphology and function of HepG2 cells. HepG2 cells cultured under 3% O_2_ presented a decreased growth rate compared to HepG2 cells cultured under 20% O_2_ (data not shown). Additionally, 20% O_2_ cultured HepG2 cells tend to form clusters, whereas 3% O_2_ cultured HepG2 cells formed a monolayer of cells (Fig. 2a). To evaluate the effect of oxygen tension on hepatocyte function and maturity, the relative gene expression of several key hepatocyte differentiation markers was determined. Oxygen tension mildly affected gene expression, with a maximal induction of 2-fold and reduction of 8-fold compared to 20% O_2_ for the genes studied (Fig. 2b). Moreover, the activity of CYP3A4, an important enzyme in chemical metabolism, was determined. Although the differences were not statistically significant, CYP3A4 activity in untreated and stimulated cells was lower under 3% O_2_ (Fig. 2c).

We hypothesized that the availability of oxygen could influence the oxidative stress status of the cells through NRF2- and hypoxia-signaling. To test this, the relative gene expression of several NRF2- and hypoxia-markers was determined. Expression of most NRF2-genes was slightly decreased, whereas expression of the hypoxia-genes generally was slightly increased in 3% O_2_ cultured HepG2 cells (Fig. 2d). However, effects were mild with a maximal 3-fold induction or reduction, possibly reflecting the handling of the cells at atmospheric oxygen. Furthermore, the effect of oxygen tension on basal ROS production was determined by measuring the relative increase in fluorescence over 1 hour in cells cultured in serum-free medium. The resulting ROS production was highly similar between 20% and 3% O_2_ cultured HepG2 cells (Fig. 2e). Likewise, the ratio between GSH and GSSG was not significantly affected by oxygen tension (Fig. 2f). Both GSH and GSSG slightly decreased in 20% O_2_ cultured HepG2 cells, although not significantly (Fig. 2F). In conclusion, oxygen tension mildly effects HepG2 morphology, function and oxidative stress status.

### 3.2 Oxygen tension affects the potency of chemicals to induce oxidative stress

**Fig. 3:**
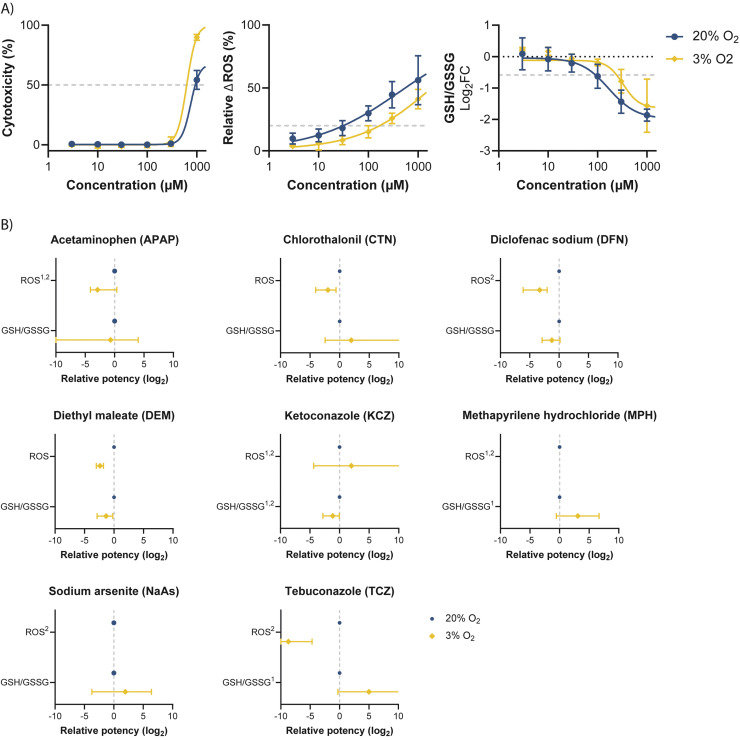
Relative potency under oxygen tension. A) Concentration-response plots for HepG2 cells exposed to diethyl maleate under different oxygen tensions. Left = cytotoxicity, middle = ROS, right = GSH/GSSG. The dashed grey line represents the set threshold for concentration exclusion (left) or relative potency calculations (middle and right). B) Relative potency of chemicals to induce 20% ROS relative to the positive control and to reduce the GSH/GSSG ratio by 33% compared to the solvent control. Data are represented as the median and 90% bootstrap percentile interval, based on 1,000 bootstraps with stratified resampling. 1 = extrapolation of the reference condition (20% O_2_), 2 = extrapolation of the test condition (3% O_2_).

Next, we assessed the influence of oxygen tension on chemically-induced effects on oxidative stress (Fig. 3a and supplementary Fig. S1). For ROS production following 1 h of chemical exposure, the potency was mostly reduced for lower oxygen tension (Fig. 3b). For two chemicals (MPH and NaAs), no relative potency could be determined as the data could not be fit using a log-logistic function. Five of the six remaining chemicals (APAP, CTN, DFN, DEM and TCZ) showed a reduced potency for cells cultured at 3% O_2_ compared to 20% O_2_. Only KCZ was calculated to have a higher potency under 3% O_2_, yet for both conditions the response did not reach the threshold for relative potency determination and results should thus be interpreted with caution (supplementary Fig. S1). Notably, under 3% O_2_ only CTN and DEM reached the set threshold for ROS production (supplementary figure S1). These results indicate that, in general, levels of chemically-induced ROS production are higher under atmospheric oxygen tension, which could lead to an overestimation of the effects that would occur under physiological conditions.

To also capture a more sustained oxidative stress response, the GSH/GSSG ratio was determined after 24 hours of chemical exposure. Half of the chemicals (APAP, DFN, DEM and KCZ) showed reduced potency, although the effect of oxygen tension on the potency of APAP to reduce the GSH/GSSG ratio was virtually none (Fig. 3b). Moreover, results for KCZ should be interpreted with caution, as neither cells cultured at 20% O_2_ nor 3% O_2_ reached the threshold for GSH/GSSG perturbation; potency calculations are thus based on extrapolation (supplementary figure S1). The other half of the chemicals (CTN, MPH, NaAs and TCZ) had increased potency to perturb the GSH/GSSG ratio under 3% O_2_ (Fig. 3b). For CTN and TCZ, the relative potencies for ROS induction and GSH/GSSG ratio perturbation are contradictory, which could be reflective of a delayed effect of these chemicals on the cellular redox status. Overall, these results indicate that the effect of oxygen tension on the GSH/GSSG ratio is chemical dependent. Moreover, the combined results on oxidative stress highlight the importance of considering physiological oxygen tension in assessing the potency of oxidative stressors.

### 3.3 Maturation strongly affects the phenotype of HepG2 cells

**Fig. 4:**
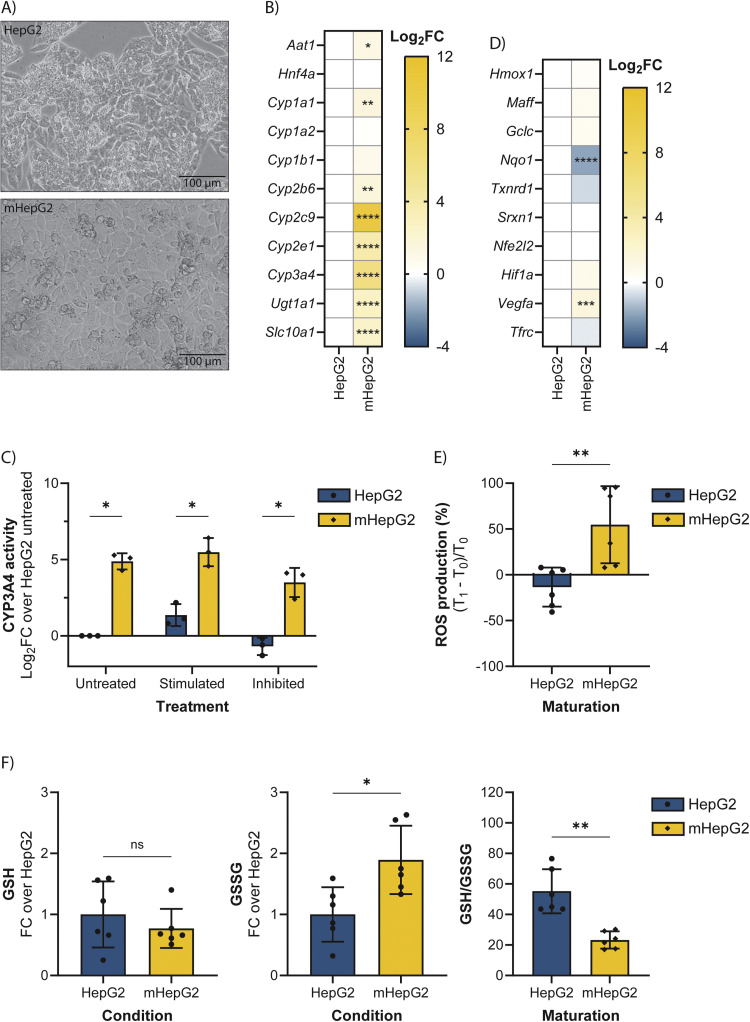
Maturation influences HepG2 phenotype. A) Representative brightfield images of non-maturated (left) and maturated (right) HepG2 cells using a 10X magnification. Scale bar = 100 µm. B) Relative gene expression (log_2_) changes of hepatocyte markers in mHepG2 cells compared to HepG2 cells. N = 3 for HepG2 and N = 6 for mHepG2. C) CYP3A4 activity in HepG2 cells and mHepG2 cells. Stimulated cells were treated with 50 µM dexamethasone for 24 hours. Inhibited cells were co-incubated with 1 µM ketoconazole. Log2-fold change compared to untreated HepG2 cells. N = 3 D) Relative gene expression (log_2_) changes of NRF2- and hypoxia-pathway related genes in mHepG2 cells compared to HepG2 cells. N = 3 for HepG2 and N = 6 for mHepG2. E) ROS production over 1 hour in non-maturated and maturated HepG2 cells. F) GSH, GSSG and GSH/GSSG ratio in non-maturated and maturated HepG2 cells. For GSH and GSSG, fold change compared to non-maturated HepG2 cells. Statistical significance was determined using a Welch’s T-test: ** < 0.0021. N = 6. Data are represented as mean ± standard deviation. For panels B, D: Statistical analysis was performed using a two-way ANOVA followed by post-hoc unpaired t-tests corrected for multiple testing using the false discovery rate (FDR) method: * < 0.05, **** < 0.0001.

Similarly as for oxygen tension, we first assessed the effect of maturation on HepG2 morphology and functioning before testing chemical exposures. In line with published literature, mHepG2 cells stopped proliferating during the differentiation and presented a monolayer of cobblestone-like cells (Boon et al., 2020; Ter Braak et al., 2022), whereas non-maturated HepG2 cells presented a more stretched morphology (Fig. 4a). To assess the relative differentiation level, gene expression of several key hepatocyte differentiation markers was determined next. Maturated cells showed strong upregulation of most of the tested hepatocyte differentiation markers (Fig. 4b). Moreover, the activity of CYP3A4 significantly increased in all three conditions with mHepG2 cells (Fig. 4c).

Given the inhibition of HepG2 proliferation and subsequent formation of a dense monolayer, we expected differences in the oxidative stress status of the maturated cells. However, basal level gene expression changes of several NRF2- and hypoxia-markers were small (Fig. 4d). Interestingly, mHepG2 cells showed a similar pattern as 3% O_2_ cultured HepG2 cells (compare Fig. 2, Fig. 4). Next, the effect of maturation on basal ROS production was determined by measuring the relative increase in fluorescence over 1 hour in cells cultured in serum-free medium. Basal ROS production significantly increased in mHepG2 cells (Fig. 4e). In line with this, the GSH/GSSG ratio significantly decreased in mHepG2 cells indicating that these cells have a lower redox buffering capacity (Fig. 4f). The decreased GSH/GSSG ratio resulted from a significant increase in GSSG in mHepG2 cells, while GSH remained virtually identical (Fig. 4f). In conclusion, maturation strongly affects HepG2 phenotype and the cell’s oxidative stress status.

### 3.4 Maturation impacts chemically-induced oxidative stress

**Fig. 5:**
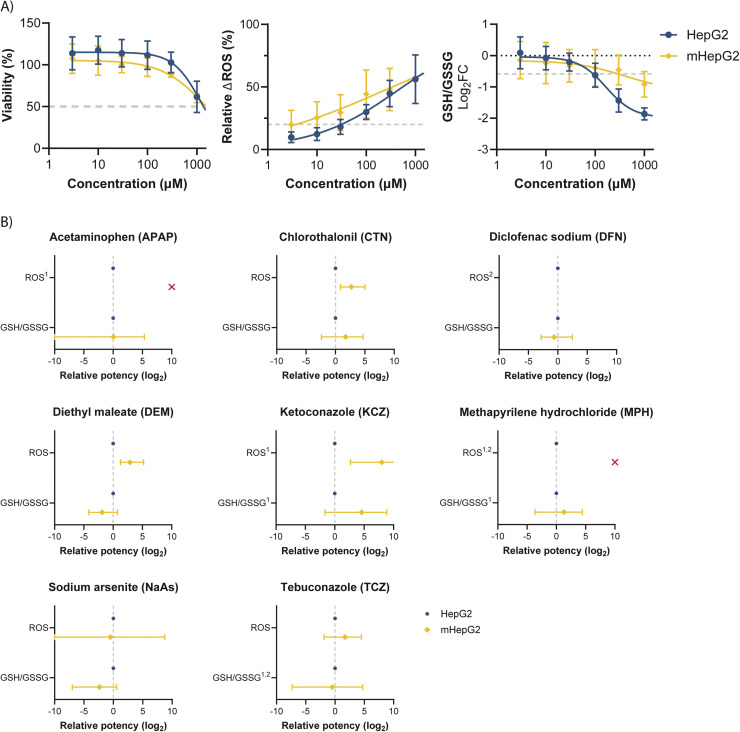
Relative potency upon maturation. A) Concentration-response plots for non-maturated and maturated HepG2 cells exposed to diethyl maleate. Left = viability, middle = ROS, right = GSH/GSSG. The dashed grey line represents the set threshold for concentration exclusion (left) or relative potency calculations (middle and right). B) Relative potency of chemicals to induce 20% ROS relative to the positive control and to reduce the GSH/GSSG ratio by 33% compared to the solvent control. Data are represented as the median and 90% bootstrap percentile interval, based on 1,000 bootstraps with stratified resampling. A red cross represents a log2-transformed relative potency > 10. 1 = extrapolation of the reference condition (HepG2 cells), 2 = extrapolation of the test condition (mHepG2 cells).

Next, we assessed the effect of cellular maturation on oxidative stress following chemical exposure (Fig. 5a and supplementary figure S2). The potency of six chemicals (APAP, CTN, DEM, KCZ, MPH and TCZ) to induce ROS formation increased in mHepG2 cells (Fig. 5b). Given the lower GSH/GSSG ratio in untreated mHepG2 cells (Fig. 4f), these cells are expected to be more susceptible to oxidative stress. Results for MPH should be interpreted with caution, as the threshold for potency calculations was not reached for both HepG2 and mHepG2 cells (supplementary figure S2). No relative potency could be determined for DFN as the data could not be fit using a log-logistic function. Nevertheless, DFN’s ROS inducing potency is considered to be reduced in mHepG2 cells as the HepG2 data did follow a concentration-response pattern and reached the set threshold for relative potency calculations (supplementary figure S2). Given the small observed differences in the concentration-response plots and the wide bootstrap interval, the potency of NaAs was considered similar between HepG2 and mHepG2 cells (supplementary figure S2). These results seemingly indicate that mHepG2 cells are more sensitive to chemically-induced ROS production for most of the tested chemicals.

As we aimed to study a more sustained oxidative stress response, the GSH/GSSG ratio was determined in mHepG2 cells after 24 hours of chemical exposure. No substantial change in potency was observed for APAP, DFN and TCZ based on the GSH/GSSG ratio (Fig. 5b). However, this observation is questionable for TCZ as neither condition met the set threshold for potency calculations (supplementary figure S2). Given the higher potency of DEM to induce ROS formation and its lower potency to perturb the GSH/GSSG ratio (Fig. 5, Fig. 5), mHepG2 cells appear capable or recovering from the immediate effect of DEM exposure. Similarly, the differences in relative potency between ROS production and GSH/GSSG ratio perturbation observed for APAP, NaAs and TCZ suggest a comparable recovery ability of mHepG2 cells (Fig. 5b). For CTN, KCZ and MPH an increased potency was seen upon cellular maturation, which overlaps with ROS production effects. Taken together, these results highlight the need for considering the representativity of the target organ when assessing chemically-induced oxidative stress, as for seven of the eight tested chemicals at least one measure of oxidative stress potency substantially changed upon cellular maturation.

### 3.5 Maturated HepG2 cells can be exposed over a prolonged period

**Fig. 6:**
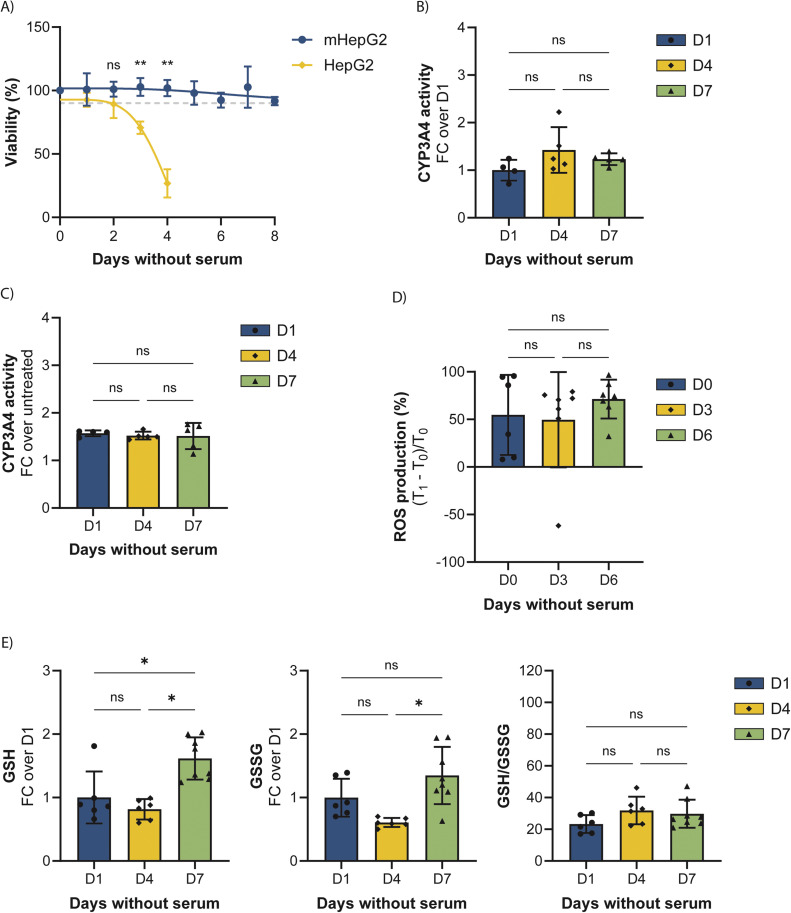
Maturated HepG2 cells are stable in absence of serum over a prolonged period. A) Serum-free survival of non-maturated and maturated HepG2 cells as determined by mitochondrial activity. N = 3. B) CYP3A4 activity over time in untreated mHepG2 cells, expressed as fold change compared to day 1. N = 4-5. C) CYP3A4 activity over time in mHepG2 cells stimulated with 50 µM dexamethasone for 24 hours, expressed as fold change over untreated mHepG2 cells. N = 4-5. D) ROS production over 1 hour in mHepG2 cells cultured in absence of serum. E) GSH (left), GSSG (middle) and GSH/GSSG ratio (right) in in mHepG2 cells cultured in absence of serum. For GSH and GSSG, fold change compared to mHepG2 cells on day 1. N = 6-8. Data are represented as mean ± standard deviation. Statistical analysis was performed using a one-way ANOVA followed by post-hoc unpaired t-tests corrected for multiple testing using the false discovery rate (FDR) method: ** < 0.01, *** < 0.001.

Since mHepG2 cells lose their proliferating capacity, these cells can be maintained longer (Boon et al., 2020). To assess if mHepG2 cells could also be exposed to chemicals for multiple days, viability of the cells in absence of serum was determined. mHepG2 cells could be cultured in absence of serum for up to eight days without cell viability declining below 90%, whereas non-maturated HepG2 cells presented a decline in cell viability already after three days in absence of serum (Fig. 6a). Additionally, we assessed if the CYP3A4 activity remained stable in the absence of serum by determining both baseline and induced CYP3A4 activity on different days after switching to serum-free medium. No differences in either baseline CYP3A4 activity (Fig. 6b) nor chemically-induced CYP3A4 activity were observed between 1, 4 and 7 days in serum-free culture (Fig. 6c). Next, we determined the effect of serum-free culture on basal ROS production, similarly as for oxygen tension and maturation. ROS production remained stable during serum-free culture (Fig. 6d). Likewise, the ratio between GSH and GSSG did not change over time without serum (Fig. 6e). At day 7, however, we observed a statistically significant increase in GSSG compared to day 1 that was not reflected in the GSH/GSSG ratio due to a concurrent, increase in GSH at day 7 (Fig. 6e). In conclusion, maturated HepG2 cells can be exposed to chemicals over a prolonged period, during which the absence of serum does not significantly affect the cell’s phenotype nor its oxidative stress status.

### 3.6 Chemically-induced oxidative stress increases upon repeated exposure

**Fig. 7:**
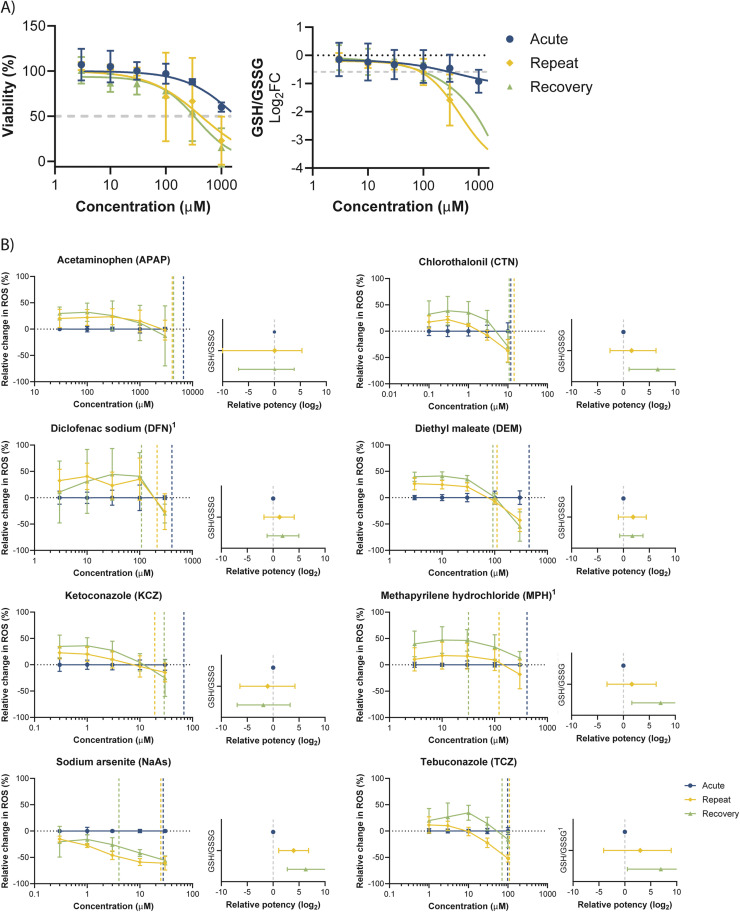
Relative potency upon repeated exposure. A) Concentration-response plots for maturated HepG2 cells exposed to diethyl maleate for 24 hrs (acute), 4×24hrs (repeat) or 4×24hrs with 24 hrs recovery in between (recovery). Left = viability, right = GSH/GSSG. The dashed grey line represents the set threshold for concentration exclusion (left) or relative potency calculations (right). B) Relative percentage change in ROS induction in repeat and recovery treated cells compared to acute treated cells (right). Dashed lines represent the concentrations that reduced viability by 20% in the respective exposure scenario. Data are represented as the mean ± standard deviation. Relative potency of chemicals to reduce the GSH/GSSG ratio by 33% compared to the solvent control (left). Data are represented as the median and 90% bootstrap percentile interval, based on 1,000 bootstraps with stratified resampling. 1 = extrapolation of the reference condition (acute), 2 = extrapolation of the test condition (repeat or recovery).

We continued to study the effect of repeated exposure with and without recovery periods in between exposures on oxidative stress (Fig. 1 for exposure scenarios, Fig. 7a and supplementary figure S3). The potency to induce ROS increased for all but one chemical (NaAs) upon repeated exposure with or without recovery periods (Fig. 7b). Interestingly, the increased potential to induce ROS was only observed for the lower concentrations tested. For those concentrations that reached the threshold for either ROS or GSH/GSSG in acutely exposed cells (supplementary figure S3), a reduced potential to induce ROS upon repeated exposure was observed (Fig. 7b). This reduction occurred at concentrations lower than those that caused 20% cell death for most of the chemicals (Fig. 7b dashed lines). Interestingly, most of the tested chemicals (CTN, DEM, KCZ, MPH, NaAs and TCZ) showed a higher relative change in ROS upon repeated exposure with recovery periods, although error bars are often overlapping (Fig. 7b).

The GSH/GSSG ratio was measured at the end of each exposure scenario (Fig. 7a and supplementary figure S3). For six chemicals (CTN, DFN, DEM, MPH, NaAs and TCZ) the potency to perturb the GSH/GSSG ratio increased in cells that were repeatedly exposed (Fig. 7b). Only APAP and KCZ showed a similar or decrease potency between the acute and repeat or recovery scenario. This indicates that the cells can potentially recover from the effects induced by these chemicals, as increased ROS production was observed (Fig. 7b and supplementary figure S3). Notably, effects on the GSH/GSSG ratio were observed at either lower or similar concentrations that induced approximately 20% cell death (supplementary figure S3), indicating that these results are not the effect of excessive cell death. Again, most of the tested chemicals (CTN, DFN, MPH, NaAs and TCZ) showed higher sensitivity to GSH/GSSG perturbation upon repeated exposure with recovery periods (Fig. 7b). Taken together, these results indicate that, for most chemicals tested, toxic effects are exacerbated upon repeated exposure independent of cytotoxicity.

## 4 Discussion

In this study, we evaluated how oxygen tension, cellular maturation, and exposure frequency influence chemically-induced oxidative stress in HepG2 cells. While a lower oxygen tension (3% O_2_) only mildly affected HepG2 phenotype and basal oxidative stress status, it had a substantial, but chemical-dependent impact on oxidative stress markers. Cellular maturation significantly altered HepG2 phenotype, increasing susceptibility to most of the tested chemicals. Furthermore, maturated HepG2 cells maintained stable viability, metabolic activity and basal oxidative stress status during prolonged, serum-free culture, allowing for repeated chemical exposure. Repeated chemical exposure, especially with recovery periods, intensified oxidative stress potency. Overall, these findings emphasize the necessity of considering oxygen tension, cellular maturation, and exposure regimen when interpreting *in vitro* toxicity data for assessing real-life impact of chemical stressors.

In cells cultured under more physiological oxygen tension (3% O_2_), representing pericentral hepatocytes, chemicals generally displayed lower potency to induce ROS, likely due to the reduced availability of oxygen. These results are in line with the reduced NRF2 target gene expression and decreased ROS production in HepG2 cells cultured at 3% O_2_, although the latter was not statistically significant. Several other studies also found a protective effect of hypoxia on ROS formation (Guo et al., 2017; Ma et al., 2017). For the GSH/GSSG ratio, we observed an increased potency for some chemicals, but a reduced potency for others. The potency for DEM, a classic GSH depleting chemical (Comporti et al., 1991), was reduced, potentially reflecting the higher baseline GSH/GSSG ratio in hypoxic cells. On the other hand, TCZ for example, a mitochondrial toxicant (Petricca et al., 2019) exhibited increased potency to perturb the GSH/GSSG ratio in HepG2 cells cultured at 3% O_2_. This may be attributed to hypoxia-induced impairment of the mitochondrial electron transport chain (Fuhrmann & Brune, 2017; Koziel & Jarmuszkiewicz, 2017), making the cells more vulnerable to mitochondrial disruption. Although this did not apply to all tested chemicals reported to affect mitochondrial function. An alternative explanation could be related to the cellular retention of these chemicals: DEM is expected to be short-lived intracellularly because of its hydrophilicity and rapid reaction with GSH, whereas TCZ is expected to persist because it is lipophilic. Nonetheless, according to our results conventional culture conditions (20% O_2_) may result in over- or underestimation of chemically-induced oxidative stress responses, possibly dependent on the chemical’s mode of action or cellular retention and chosen readout.

Metabolic maturation of HepG2 cells profoundly influenced their redox status and response to chemical stressors. We found that for six of the eight tested chemicals the potency to induce oxidative stress, either based on ROS formation of GSH/GSSG perturbation, was substantially increased in mHepG2 cells. These results reflect the significantly higher baseline ROS and lower baseline GSH/GSSG ratio in maturated cells. However, for DEM we observed a higher ROS induction potency but a lower GSH/GSSG perturbation potency. This could be an indication that mHepG2 cells, like hepatocytes (Gerets et al., 2012; Sison-Young et al., 2015; Soderdahl et al., 2007), have a greater capacity to restore the GSH/GSSG ratio if there is no other mechanism contributing to oxidative stress or if the chemical has a transient effect. Another important difference between maturated and non-maturated HepG2 cells to consider is their metabolic capacity. We showed that mHepG2 have elevated gene expression and activity of some phase I metabolic enzymes, in line with published literature (Boon et al., 2020; Pozo Garcia et al., 2025). Observed differences could thus result from bioactivation of chemicals in mHepG2 cells. Yet, literature also indicates that mHepG2 cells have improved activity of some phase II enzymes compared to HepG2 cells (Pozo Garcia et al., 2025), which is in line with our finding of increased *Ugt1a1* expression in mHepG2 cells. This means that although mHepG2 cells have an improved capacity to bioactivate chemicals, likely this test system also has a better detoxification capacity. Furthermore, we found increased expression of *Slc10a1*, a transporter involved in chemical uptake, in mHepG2 cells. This hypothesis was further substantiated upon comparison of previously generated transcriptomic data of PHH, HepG2 and mHepG2 cells. In general, mHepG2 cells showed higher gene expression of metabolism- and transporter-genes compared to HepG2 cells although the expression is still substantially lower than in PHH (supplementary figure S4A). As gene expression cannot directly be correlated to protein or enzyme activity, these results should be interpreted cautiously. Given these findings, it would be interesting to measure intracellular chemical concentrations in both non-maturated and maturated HepG2 cells, as well as for the exposure scenarios evaluated, to get a better understanding of potency differences. Nevertheless, the differences in chemical potency on oxidative stress urge for considering organ representativity and metabolic capacity in chemical hazard characterization.

Interestingly, in our repeat and recovery exposure scenarios, we observed that lower chemical concentrations led to increased ROS, while higher concentrations, which induced acute ROS or perturbed GSH/GSSG, resulted in lower fluorescent signal (supplementary figure S3 and Fig. 7b). We hypothesized that this conflicting effect is due to the properties of the fluorescent probe used for ROS quantification. The probe becomes fluorescent upon oxidation by ROS, but once oxidized, its fluorescence cannot increase further, regardless of additional ROS. Therefore, at high ROS concentrations, the probe signal may reach saturation, limiting our ability to detect further increases in ROS formation. Moreover, our protocol for ROS production quantification using the DCFH probe is not suited for repeated exposure since baseline fluorescence cannot be accurately established after prior exposures. With our adjusted analysis we were able to compare ROS in acutely exposure cells with the repeated scenarios, but not ROS production during one hour of exposure. Time-response measurements, for instance using the SRXN1 fluorescent reporter from ToxProfiler (Ter Braak et al., 2024), could provide dynamic insights into NRF2 activity throughout exposure and recovery.

Although the DCFH probe allowed us to study ROS formation over one hour of chemical exposure, this probe has some noteworthy limitations hindering its application in a regulatory setting. First, the DCFH probe lacks ROS specificity (Kalyanaraman et al., 2012; Murphy et al., 2022). While this means the probe can be used as a marker for general ROS levels, it also means this probe cannot provide insight into the source of ROS (e.g., mitochondrial). Contrarily, previous research has shown that the DCFH probe primarily reacts with hydrogen peroxide and hydroxyl, and not superoxide radicals (Gomes et al., 2005; LeBel et al., 1992). Therefore, mitochondrial ROS might be missed in the current setup. Second, fluorescence of the probe is dependent on intracellular esterase activity (Reiniers et al., 2021). Activity of these esterases can vary substantially between cell and tissue types (Jones et al., 2013; Wang et al., 2018). Lastly, the DCFH probe has been shown to be affected by pH (Huang et al., 2016). As such, chemicals influencing the pH of cell culture media could lead to probe fluorescence without actual ROS production. Given these limitations, we believe that regulatory testing for oxidative stress inducing potential of chemicals should consist of multiple assays, preferably targeting different aspects and dynamics (e.g., ROS, GSH/GSSG ratio and NRF2-mediated gene expression).

Our results indicate that for some chemicals toxic effects are exacerbated upon recovery periods (supplementary figure S3). We hypothesized that this difference is due to dynamic changes in cellular signaling pathways. During exposure, cells predominantly activate survival signaling mechanisms, which equip them to withstand ongoing stress. However, during recovery periods, the cells shift from survival signaling to repair signaling, focusing on regeneration, which the liver is capable of (Bhushan & Apte, 2019, 2023). This transition may render the cells temporarily less prepared to manage a subsequent toxic insult, thereby increasing their sensitivity to additional exposures. In contrast, repeated exposure without recovery maintains survival signaling, which may confer greater resilience to further toxicity. Testing this hypothesis could involve transcriptomic analyses to identify key signaling pathways activated at various timepoints during exposure and recovery. Alternatively, cellular retention or chemical plastic binding could play a role. During exposure, part of the chemical could bind to the plastic and is then released during recovery with fresh medium. Of the tested chemicals, CTN, KCZ and TCZ were predicted to have some plastic binding using virtual *in vitro* distribution modelling (data not shown). These three chemicals are also expected to have higher cellular retention compared to the other chemicals based on lipophilicity. However, MPH and NaAs also showed evidence of intensified effects upon recovery.

Although maturated HepG2 cells provide an improved model over proliferating HepG2 cells, their metabolic capacity remains substantially inferior to that of primary hepatocytes (Pozo Garcia et al., 2025). Additionally, the current 2D monoculture of HepG2 cells fails to capture the complex 3D, multicellular architecture of the *in vivo* liver. Therefore, future research should focus on developing and implementing more physiologically relevant models, such as spheroids or liver microtissues that consist of a co-culture with primary hepatocytes, Kupffer cells and stellate cells in a 3D conformation. 3D models were shown to have improved metabolic capacity (Bell et al., 2018; Berger et al., 2016; Ramaiahgari et al., 2014; Stampar et al., 2020; Ter Braak et al., 2022) and to be suitable for repeated exposure scenarios (Bell et al., 2016; Proctor et al., 2017; Ramaiahgari et al., 2014; Ter Braak et al., 2022). Moreover, due to their 3D structure, these models are expected to better recapitulate the oxygen gradient, representing both pericentral and periportal hepatocytes, without causing artificial oxygen spikes upon handling of the cells inherently associated with culturing at lower oxygen. We performed an exploratory analysis of the expression of liver function, oxidative stress and hypoxia-related genes between PHH, HepG2, mHepG2, and induced pluripotent stem cell-derived hepatocyte-like cells (iPSC-HCL). Although mHepG2 showed a higher correlation with PHH compared to HepG2, iPSC-HLC outperformed mHepG2 cells on both gene sets (supplementary figure S4A-C). As such, we suggest future research to focus on the characterization of novel models and the development of suitable oxidative stress assays for 3D models. Results obtained from these complex models could then be compared with those from simpler systems to determine their value for regulatory toxicological testing. Furthermore, for next generation risk assessment workflows, tested concentrations should be based on predicted human exposure levels, determined by integrated physiologically-based kinetic (PBK) modelling, quantitative *in vitro* to *in vivo* extrapolation (qIVIVE), and virtual *in vitro* distribution modelling, to improve the human relevance of the exposure scenarios. Ultimately, adopting models that better mimic *in vivo* conditions will improve the predictive value of hazard characterization and reduce reliance on animal testing for chemical risk assessment.

## Disclaimer

This work reflects only the authors’ views, and the European Commission is not responsible for any use that may be made of the information it contains.

## Funding sources

This work was supported by the project RISK-HUNT3R: RISK assessment of chemicals integrating HUman centric Next generation Testing strategies promoting the 3Rs. RISK-HUNT3R has received funding from the European Union's Horizon 2020 research and innovation programme under grant agreement No 964537 and is part of the ASPIS cluster.

## CRediT authorship contribution statement

**Christina H.J. Veltman:** Writing – review & editing, Writing – original draft, Visualization, Investigation, Formal analysis, Conceptualization. **Jeroen L.A. Pennings:** Writing – review & editing, Supervision, Conceptualization. **Bob van de Water:** Writing – review & editing, Supervision, Funding acquisition. **Mirjam Luijten:** Writing – review & editing, Supervision, Funding acquisition, Conceptualization.

## Declaration of competing interest

The authors declare that they have no known competing financial interests or personal relationships that could have appeared to influence the work reported in this paper.

## Data Availability

The raw data that support the findings of this study are publicly available in BioStudies: HepG2 LDH data (S-BSST2269), (m)HepG2 CellTiter data (S-BSST2267), (m)HepG2 DCFH data (S-BSST2254), and (m)HepG2 GSH/GSSG data (S-BSST2258).https://www.ebi.ac.uk/biostudies/

## Appendix Supplementary materials

Image, application 1

Image, application 2
