# Supplementary material for: Impact of culture conditions on oxidative stress responses in HepG2 cells
Source: NAM J. 2026 Jul 10;2:100113. doi: 10.1016/j.namjnl.2026.100113 (PMC13400215; doi:10.1016/j.namjnl.2026.100113)
Supplement: Supplementary file 1 [file mmc1.docx]

**Impact of culture conditions on oxidative stress responses in HepG2 cells**

Christina H. J. Veltman^1,2^, Jeroen L. A. Pennings^1^, Bob van de Water^2^ & Mirjam Luijten^1,2^

*^1^Centre for Health Protection, National Institute for Public Health and the Environment (RIVM), Bilthoven, The Netherlands; ^2^Division of Cell Systems and Drug Safety, Leiden Academic Centre for Drug Research (LACDR), Leiden University, Leiden, The Netherlands*

ORCIDs: Christina H.J. Veltman 0000-0002-3975-6855; Jeroen L.A. Pennings 0000-0002-9188-6358; Bob van de Water 0000-0002-5839-2380; Mirjam Luijten 0000-0002-5277-1443

Corresponding author:

Mirjam Luijten

Centre for Health Protection, National Institute for Public Health and the Environment (RIVM), P.O. Box 1, 3720 BA Bilthoven, The Netherlands

Email: mirjam.luijten@rivm.nl

**Supplement**

*Supplementary methods. Analysis of transcriptomic count data*

Transcriptomic count data for selected genes was collected from previously performed experiments (BioStudies S-RHER322 and unpublished data; supplementary table 1). Differential gene expression was assessed for each experiment by calculating log2 fold changes relative to PHH, which served as the reference control. This analysis provided a measure of the relative expression of each gene in the liver cell models compared to PHH.


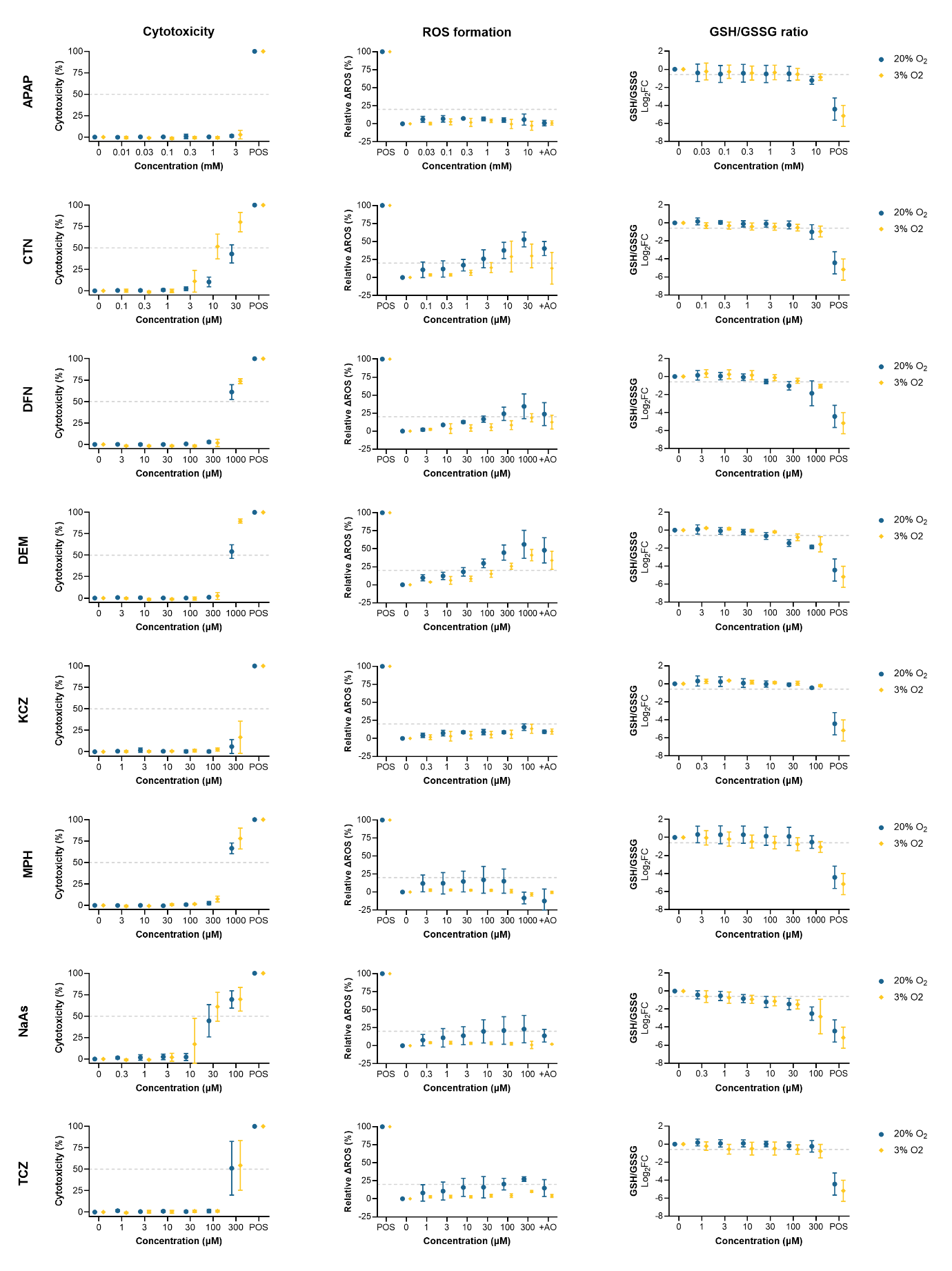


**Supplementary figure S1. Concentration-response plots for oxygen tension.** Concentration-response plots for cytotoxicity measured by LDH release (left), ROS formation (middle) and GSH/GSSG (right) per chemical for cells cultured under 20% O2 (blue circle) or 3% O2 (yellow rhombus). N = 3. Data is represented at the mean ± standard deviation. The grey dashed lines represent the threshold for concentration exclusion (left) or relative potency calculations (middle and right): 50% cytotoxicity, 20% relative ROS production compared to the positive control, and 33% decrease in GSH/GSSG ratio compared to the solvent control (0.1% DMSO). Positive controls include 1% Triton (cytotoxicity), 1 mM hydrogen peroxide (ROS) and 40 µM menadione (GSH/GSSG). 30 µM N, N’-Diphenyl-p-phenylenediamine was used as antioxidant co-treatment (+AO) for the highest tested concentration in ROS experiments.


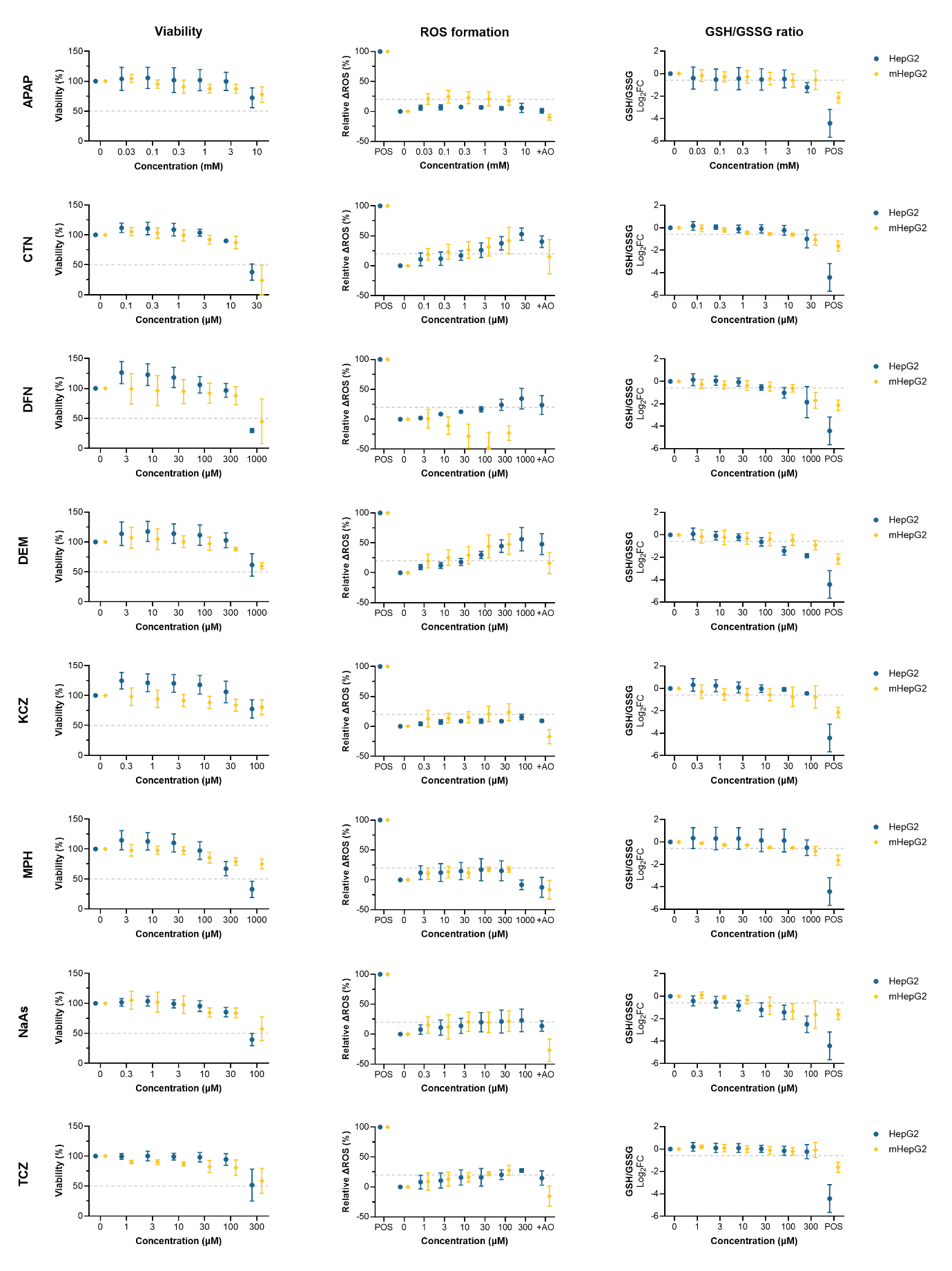


**Supplementary figure S2. Concentration-response plots for maturated HepG2.** Concentration-response plots for viability measured by protease activity (left), ROS formation (middle) and GSH/GSSG (right) per chemical for HepG2 (blue circle) or mHepG2 cells (yellow rhombus). N = 3. Data is represented at the mean ± standard deviation. The grey dashed lines represent the threshold for concentration exclusion (left) or relative potency calculations (middle and right): 50% viability, 20% relative ROS production compared to the positive control, and 33% decrease in GSH/GSSG ratio compared to the solvent control (0.1% DMSO). Positive controls 1 mM hydrogen peroxide (ROS) and 40 µM menadione (GSH/GSSG). 30 µM N, N’-Diphenyl-p-phenylenediamine was used as antioxidant co-treatment (+AO) for the highest tested concentration in ROS experiments.


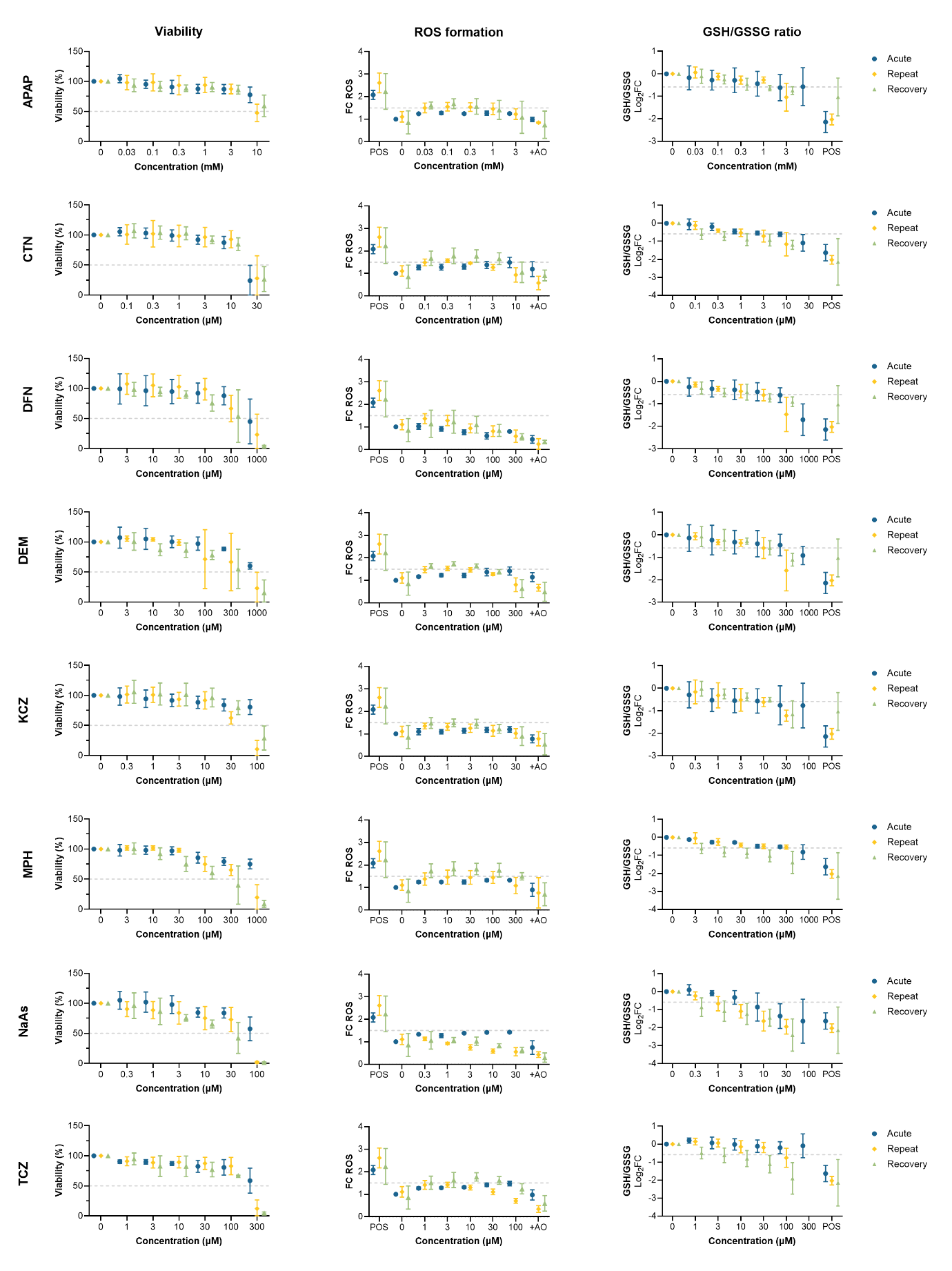


**Supplementary figure S3. Concentration-response plots for exposure scenarios.** Concentration-response plots for viability measured by protease activity (left), ROS (middle) and GSH/GSSG (right) per chemical for acute (blue circle), repeat (yellow rhombus) or recovery (green triangle) exposure. N = 3. Data is represented at the mean ± standard deviation. The grey dashed lines represent the threshold for concentration exclusion (left) or relative potency calculations (right): 50% viability and 33% decrease in GSH/GSSG ratio compared to the solvent control (0.1% DMSO). For ROS, the grey dashed line represents a 1.5-fold increase compared to the solvent control (0.1% DMSO). Positive controls include 1 mM hydrogen peroxide (ROS) and 40 µM menadione (GSH/GSSG). 30 µM N, N’-Diphenyl-p-phenylenediamine was used as antioxidant co-treatment (+AO) for the highest tested concentration in ROS experiments.


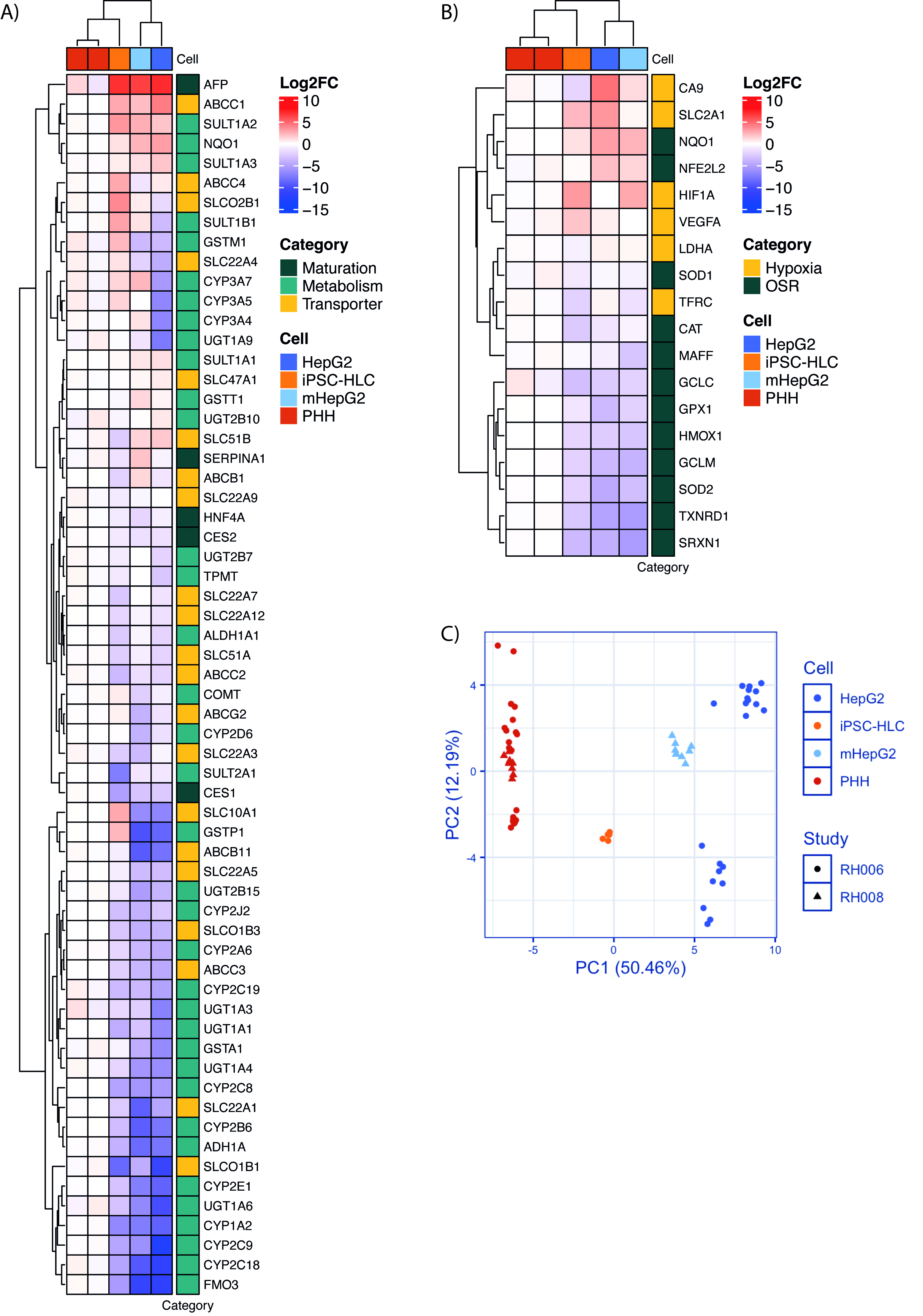


**Supplementary figure S4. Transcriptomic comparison of cellular liver models.** A) Log2 fold change in gene expression compared to PHH for a liver function gene set. B) Log2 fold change in gene expression compared to PHH for an oxidative stress- and hypoxia-related gene set. C) Principal component analysis (PCA) plot of log2-transformed counts per million for the combined gene set of liver function, oxidative stress- and hypoxia-related genes studied.
